# User perception of a new hydrophilic‐coated male urinary catheter for intermittent use

**DOI:** 10.1002/nop2.193

**Published:** 2018-09-04

**Authors:** Ingrid Koeter, Gro Stensröd, Aud Hunsbedt Nilsen, Rigmor Lund, Colette Haslam, Marianne De Sèze, Rajagopalan Sriram, John Heesakkers, the LoFric Origo study group

**Affiliations:** ^1^ Rivas Zorggroep Gorinchem The Netherlands; ^2^ Urodynamisk laboratorium Sunnaas Sykehus HF Nesoddtangen Norway; ^3^ Urologisk poliklinikk Stavanger Universitetssjukehus Stavanger Norway; ^4^ Urologisk poliklinikk Akershus Universitetssykehus HF Lørenskog Norway; ^5^ National Hospital for Neurology and Neurosurgery, Queens Square London UK; ^6^ Cabinet de Neuro‐Urologie, Pelvipérinéologie et Urodynamique Groupe Urologique de la Clinique Saint Augustin Bordeaux France; ^7^ University Hospitals Coventry and Warwickshire NHS Trust, Walsgrave Hospital Coventry UK; ^8^ Radboudumc Nijmegen The Netherlands

**Keywords:** adherence, hydrophilic, intermittent catheterization, perception, urinary catheter

## Abstract

**Aims:**

This study investigated user perception and adherence related to a hydrophilic‐coated urinary catheter (LoFric® Origo™), available for male patients who practice intermittent catheterization.

**Design:**

The study had a prospective observational design, including patients from 19 European hospitals.

**Methods:**

A total of 416 patients were eligible for the study; 179 experienced catheter users and 237 de novo. Two questionnaires were filled out, one describing background data and a second, 8 weeks later, evaluating catheter features.

**Results:**

The response rate for the second questionnaire was 88% (365 patients). Patients evaluating the new catheter showed a general satisfaction rate of 81% and 72% kept using it. The hygienic grip of the catheter was appreciated by 85% and the foldable feature by 67%. The results show that convenience, ease of use, and hygienic factors are patient‐preferred features for a urinary catheter. These factors were confirmed for the evaluated hydrophilic‐coated catheter.

## 1 INTRODUCTION

Already in 1966, intermittent catheterization was recognized as the preferred method for early bladder management of the spinal injured patient (Guttmann & Frankel, 1966). Since then, the technique (Lamin & Newman, 2016; Lapides, Diokno, Silber, & Lowe, 1972) and catheters have been developed to optimize user‐friendliness and to reduce the risk of complications such as urinary tract infections (UTI) and urethral trauma. Today, intermittent catheterization is the preferred first therapy choice in favour of indwelling catheterization (Hooton et al., 2010; Lamin & Newman, 2016; Tenke et al., 2008; Tenke, Koves, & Johansen, 2014). Most national and international guidelines, such as the European Association of Urology (EAU) Guideline on Neuro‐Urology and the European Association of Urology Nurses (EAUN), recommend intermittent catheterization as the standard treatment for patients who are unable to empty their bladder (Blok et al., 2015; Vahr et al., 2013). A well‐educated nurse specialist is often responsible for teaching intermittent catheterization (Vahr et al., 2013) and a clean (Lapides et al., 1972; Vahr et al., 2013) or aseptic technique is usually recommended (Blok et al., 2015; Vahr et al., 2013). This, together with good patient education, good patient adherence, and proper catheter material, is an essential part of making the therapy as safe and effective as intended (Vahr et al., 2013; Wyndaele, 2002, 2002 ).

There are many reported barriers associated with intermittent catheterization that can potentially reduce the positive outcomes of the therapy. As a result, there is a strong research focus on supportive healthcare professionals that can promote the therapy and specific catheters adapted to patients’ individual needs. The purpose of the current study is to investigate patients’ perception of and adherence to a newly developed hydrophilic‐coated catheter (LoFric® Origo™, Wellspect HealthCare, DENTSPLY IH AB, Sweden). A greater understanding of patients’ preferences, needs, and opinions is essential to ensure good adherence and outcome of intermittent catheterization.

## 2 BACKGROUND

Patient compliance or, more properly: “patient adherence”, is a key factor for ensuring a good clinical outcome (Vermeire, Hearnshaw, van Royen, & Denekens, 2001). Nonadherence has been identified as a major public health problem and it is associated with a significant financial burden (Vermeire et al., 2001). Several factors seem to affect adherence and two general examples are (a) shared decision‐making between a healthcare professional and patient (Vermeire et al., 2001); and (b) fitting the treatment solution into the patient's everyday life (Gold & McClung, 2006; Morris & Schulz, 1993). For intermittent catheterization, it is known that poor adherence to the therapy can cause urinary and renal complications (Vahr et al., 2013; van Achterberg, Holleman, Cobussen‐Boekhorst, Arts, & Heesakkers, 2008). The critical factors for adherence or how common it is, are still unknown (van Achterberg et al., 2008). One contributing factor to good adherence seems to be ensuring that patients are given an individualized catheter choice (Bardsley, 2016; Bermingham et al., 2013; Chick, Hunter, & Moore, 2013; Hill et al., 2013; Kelly, Spencer, & Barrett, 2014; NICE, 2012; Wilde, Brasch, & Zhang, 2011; Woodward, 2013). For this reason, understanding patients’ preferences, needs, and opinions about specific catheters and catheter features is essential if the consequences of nonadherence are to be avoided. There are a few reports available describing appreciated attributes for catheters by patients. Risk reduction of UTI, ease of insertion, and convenience (Neovius & Lundqvist, 2014; Pinder, Lloyd, Nafees, Elkin, & Marley, 2015) seem to be important features, but these findings still need to be translated into practical terms for specific catheters. Addressing barriers is another important factor in obtaining good adherence (van Achterberg et al., 2008). Many barriers to practicing intermittent catheterization exist. Some of the most commonly reported include:

- Inconvenience related to preparations (Cobussen‐Boekhorst, Hermeling, Heesakkers, & van Gaal, 2016)
- Access to bathrooms (Bolinger & Engberg, 2013; Seth, Haslam, & Panicker, 2014; Wilde et al., 2011)
- Age (Cobussen‐Boekhorst, Beekman, et al., 2016)
- Overall knowledge, fears, and understanding of the concept (van Achterberg et al., 2008).

Some of these barriers can be addressed by healthcare professionals and their communication skills and attitudes are instrumental in promoting confidence for patients and enabling long‐term adherence to the therapy (Vahr et al., 2013). Some of the barriers can be addressed by catheter choice and imply that a convenient catheter may improve adherence (Bennett, 2002). Awareness and knowledge among healthcare professionals is, however, key to implementing the use of intermittent catheterization by specific catheters in the everyday situation (Cobussen‐Boekhorst, Beekman, et al., 2016; Cobussen‐Boekhorst, Hermeling, et al., 2016; van Achterberg et al., 2008).

Specific catheter types for intermittent use are debated. A recent Cochrane review concluded that solid evidence for the superiority of a specific catheter or technique is lacking and that catheter choice will be dependent on personal preference, cost, portability, and ease of use (Prieto, Murphy, Moore, & Fader, 2014, 2015). Previous work has shown that catheter features and preferences can improve patients’ quality of life related to intermittent catheterization (Chartier‐Kastler et al., 2013) and that is why the current study focuses on assessing user perception and adherence. The current catheter investigated is a hydrophilic‐coated catheter. Hydrophilic catheters were developed in the early 1980s to prevent long‐term complications associated with repeated catheterizations with uncoated catheters (Perrouin‐Verbe et al., 1995; Wyndaele & Maes, 1990). Hydrophilic catheters are known to be easy to use and comfortable (Chartier‐Kastler & Denys, 2011; Seth et al., 2014) but specific catheter features described by patients are lacking. Many patients prefer hydrophilic catheters when they have the choice (Chartier‐Kastler & Denys, 2011). Hydrophilic catheters have been reported to prevent urethral trauma (Chartier‐Kastler & Denys, 2011; Hedlund, Hjelmas, Jonsson, Klarskov, & Talja, 2001; Li, Ye, Ruan, Yang, & Zhang, 2013; Vahr et al., 2013) and reduce the risk of UTIs (Cardenas & Hoffman, 2009; Cardenas et al., 2011; Chartier‐Kastler & Denys, 2011; De Ridder et al., 2005; Li et al., 2013; Tenke et al., 2014; Vapnek, Maynard, & Kim, 2003; Woodbury, Hayes, & Askes, 2008), also after long‐term use (Bakke, Digranes, & Hoisaeter, 1997; Hakansson, Neovius, Norrback, Svensson, & Lundqvist, 2015; Waller, Jonsson, Norlen, & Sullivan, 1995). As a result, hydrophilic‐coated catheters have been identified as a cost‐effective contributor in UTI prevention, although it is recognized that catheter choice should not solely be based on UTI risk as patient comfort and ease of use are important criteria (Lamin & Newman, 2016).

## 3 THE STUDY

### 3.1 Design

This was a prospective study, observing real‐life use of intermittent catheterization in a cohort of male patients. The inclusion criteria were male patients, 18 years and older, who practiced daily intermittent catheterization with LoFric Origo, a hydrophilic‐coated catheter. LoFric Origo is a male, ready‐to‐use catheter that is foldable into pocket size and has an adjustable insertion grip to enable no‐touch catheterization. The packaging has a discrete design and doubles as a hygienic disposal pouch.

### 3.2 Method

Patients attending or newly admitted at any of the 19 participating European clinics/hospitals (two in Switzerland, three in Belgium, two in the Netherlands, four in Norway, four in France, and four in the UK) were asked to participate in the study. Included patients were asked to fill out two questionnaires. The selection of questions in the patient‐reported questionnaires were based on previous findings from studies of similar catheters (e.g., (Johansson et al., 2013)), experiences from product development from the manufacturer and clinical expertise. That is, during questionnaire development, content validity was considered by clinical experts part of the LoFric Origo study group. The views and opinions of urology nurses, urotherapists, continence advisors, urologists, and neuro‐urologists were considered. Internal validity and reliability of the questionnaires were not formally tested but the design reflected consistency with previous experiences from clinical studies and product development. The first questionnaire collected background data (e.g., reason for therapy, hand function, urethral sensitivity) and was filled out when attending the clinic. Specific data (e.g., catheter type, time on catheter, catheterization frequency, and perception of handling, satisfaction and convenience) were collected for patients with previous experience with intermittent catheterization. This was not applicable for newly admitted patients. The second questionnaire collected data on patient preferences and specific catheter features (e.g., perception of handling, satisfaction, convenience) and captured if the patients still used the catheter or any reasons for discontinuation (i.e., adherence). The second questionnaire also contained a list of 19 commonly reported catheter attributes (e.g., easy to open, discrete design, ready to use) and patients were asked to identify and list three features that best/least described the evaluated catheter. The list was identified based on clinical expertise and current findings about important catheter attributes and barriers to intermittent catheterization (described in the background section). The second questionnaire was filled out in the patients’ home‐setting 8 weeks after the first questionnaire and clinical visit. Full questionnaires and the specific questions are found in supplementary material. Data were collected between February 2013 and March 2015.

### 3.3 Analysis

Descriptive statistics were used to analyse epidemiological data, that is, number of patients (*N*), mean, standard deviation (*SD*) for continuous data, and frequencies and percentages for categorical data. Primary analyses were conducted on the complete data from all countries. Missing data were not replaced or estimated, resulting in percentages based on the actual numbers and varying total N. Ordinal categorical data based on a 5‐grade scale with one neutral and two positive/negative answers were transformed into a three‐grade scale (i.e., positive, neutral, negative) when presented. For example, patients who answered either “completely satisfied” or “satisfied” were combined to describe “satisfaction”. Patients who answered either “not satisfied” or “not satisfied at all” were combined to describe “not satisfied”. All categories were considered and used in statistical testing. The descriptive and noninterventional nature of the research limits the use of a formal sample size estimation and inclusion was made in an arbitrary manner with the goal of covering a sample between 160‐600 patients. Statistical tests, using nonparametric tests and with *p*‐values below 0.05 considered as statistically significant, were performed when appropriate. This included the Wilcoxon rank sum test for exploring differences between unrelated subgroups (i.e., adherent vs. nonadherent patients or new vs. experienced users) and the McNemar test to compare the results between paired observations (i.e., previous catheter experience vs. hydrophilic‐coated catheter). All analyses were exploratory and hypothesis generating for further research.

### 3.4 Ethics

The study was registered at clinicaltrials.gov with the identifier NCT01796587 and reviewed and/or approved by applicable ethics committees. That is:

- Commissie Medische Ethiek Van Se Universitaire ZiekenHuizen Kuleuven — Leuven, Belgium (Belgisch Nummer B322201318122)
- Commission cantonale valasanne d'ethique médicale — Sion, Switzerland (CCVEM 011/13)
- Ethikkommission des Kantons Luzern — Luzern, Switzerland (Ref. Nr. EK:13,048), Commissie Mensgebonden Onderzoek — Nijmegen, the Netherlands (Registratienummer: 2012/504)
- Toetsingscommissie Patiëntgebonden onderzoek — Gorinchem, the Netherlands (CH/ds/13–249)
- Regionale komiteer for medisinsk og helsefaglig forskningsetikk (REK sør‐øst) — Oslo, Norway (Ref: 2013/187)
- NRES Committee London, City & East — Bristol, UK (No approval required)
- Commission Nationale de I'Infomatique et des Libertés (CNiL) — Paris, France (N/Réf.: EGY/VCS/AR132977, Décision DR‐2013–135)
- Comité consultative sur le traitement de l'informatyion en matiére de recherché dans le domaine de la santé (CCTRIS)/Ministére De l'Enseignement Supérieur Et De La Recherche – Paris, France (DGRI CCTRIS MG/CPᵒ13.053/Dossier no 13.080).

Every patient had given informed consent before participation. The catheter choice and prescription was made before the decision to participate in the study and no free catheter samples were provided in addition to those which were part of standard care. Payment and reimbursement for catheters followed standard routines of the country.

## 4 RESULTS

**Figure 1:**
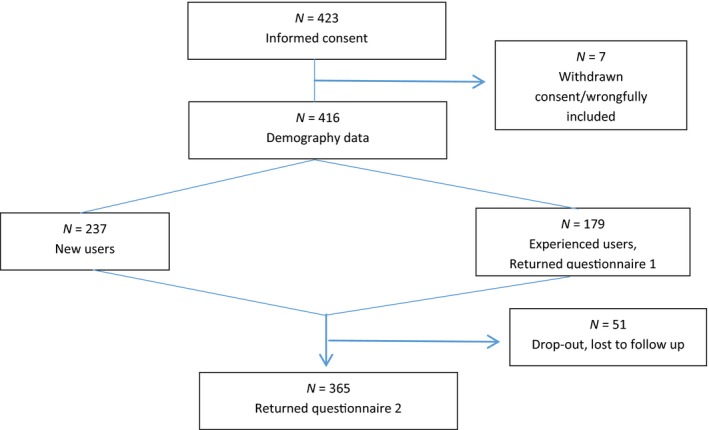
Patient flow

A total of 423 patients agreed to participate in the study but background data were only available for 416 patients (Table 1 and Figure 1). That is, seven patients were either wrongfully included or withdrew their consent, leaving 416 patients with demography data. Of the 416 patients, 179 had previous experience with catheter use and intermittent catheterization (Table 2 & Figure 1). A total of 365 patients returned the second questionnaire after 8 week's use of the hydrophilic catheter, resulting in a response rate of 88% (Table 2 & Figure 1). Figure 1 summarizes the patient flow in the study. The studied population reflected a wide range of patients with the common need for daily intermittent catheterization. Background data are found in Table 1.

**Table 1:** Background data

Background data NB: Only male patients included		
Age (*N* = 418)		
Mean (*SD*)	58 (16)	
Median (Min‐Max)	63 (18–87)	
Main reason for intermittent catheterization (*N* = 416)	*N*	%
Brain and/or spinal cord disease (e.g., multiple sclerosis, myelitis, tumor, cyst)	55	13%
Neural tube defects (e.g., spina bifida)	9	2%
Spinal cord injury, paraplegia	61	15%
Spinal cord injury, tetraplegia	13	3%
Bladder dysfunction (e.g., underactive detrusor, overactive bladder)	113	27%
Bladder outlet obstruction (e.g., prostate hyperplasia)	117	28%
Postsurgical condition (e.g., neobladder, orthotopic bladder substitutes)	10	2%
Other (e.g., strictures)	38	9%
Time on intermittent catheterization (*N* = 416)		
New user	237	57%
<3 months	22	5%
3 months–1 year	13	3%
1–3 years	40	10%
>3 years	104	25%
Hand function (*N* = 416)		
Normal	369	89%
Slightly reduced	35	8%
Considerable reduced	12	3%
Urethral sensation (*N* = 416)		
Normal	323	78%
Reduced	60	14%
No sensitivity	33	8%

**Table 2:** Catheter characteristics

	Previous catheter (*N* = 179)		LoFric Origo (*N* = 365)		*p*‐value
Time on previous catheter					
<3 months	27	15%	NA		
3 months–1 year	22	12%	NA		
1–3 years	45	25%	NA		
>3 years	85	47%	NA		
Daily catheterization frequency					
1 time per day	13	7%	48^a^	16%	
2 times per day	16	9%	61^a^	20%	
3 times per day	17	9%	32^a^	11%	
4 times per day	24	13%	55^a^	18%	
5 times per day	55	31%	61^a^	20%	
6 times per day	35	20%	30^a^	10%	
7 times per day	10	6%	7^a^	2%	
>7 times per day	9	5%	8^a^	3%	
Position during catheterization					
Sitting	61	34%	86^b^	24%	
Standing	111	62%	264^b^	74%	
Lying down	7	4%	9^b^	3%	
Patients satisfaction					0.5657^k^
Completely satisfied/satisfied	141	79%	289^b^	81%	
Neutral	29	16%	32^b^	9%	
Not satisfied/not satisfied at all	9	5%	38^b^	11%	
Handling at insertion					0.0886^k^
Very easy/easy	139	78%	300^c^	83%	
Neutral	29	16%	42^c^	12%	
Difficult/very difficult	11	6%	18^c^	5%	
Touching coating during catheter insertion					
Yes	57	32%	NA		
No, I use enclosed insertion aid	52	29%	NA		
No, I use separate insertion device	16	9%	NA		
No, I hold connector	54	30%	NA		
Insertion grip easy to use					
Yes	NA		291^b^	81%	
No	NA		29^b^	8%	
Do not use	NA		39^b^	11%	
Handling at withdrawal					0.3261^k^
Very easy/easy	164	92%	332^c^	92%	
Neutral	13	7%	21^c^	6%	
Difficult/very difficult	2	1%	7^c^	2%	
Disposal					1.0000^k^
Very easy/easy	123^d^	69%	281^c^	78%	
Neutral	33^d^	19%	53^c^	15%	
Difficult/very difficult	22^d^	12%	26^c^	7%	
Practical at home	150	86%	281^e^	82%	0.4705^k^
Practical out of home	111	64%	231^e^	66%	1.0000^k^
Additional bladder management					
Spontaneously voiding	56	31%	162^f^	47%	
External compression	7	4%	5^f^	1%	
Other (e.g., indwelling catheter, pads)	7	4%	10^f^	3%	
Incontinence between catheterizations	67^g^	37%	76^h^	21%	
Self‐catheterization	174^i^	98%	347^j^	98%	

Note

NA = Not applicable.

^a^ *N* = 302. ^b^ *N* = 359. ^c^ *N* = 360. ^d^ *N* = 178. ^e^Not all patients were able to answer this question. That is, *N* = 174 (home setting), *N* = 173 (out of home) for previous catheter and *N* = 342 (home setting) and *N* = 350 (out of home) for LoFric Origo. ^f^ *N* = 343. ^g^Among those who experienced incontinence, 61% (*N* = 41) emptied their bladder five to seven times daily, 16% (*N* = 11) three to four times daily and 22% (*N* = 15) one to two times daily. ^h^Among those who experienced incontinence, 42% (*N* = 27) emptied their bladder five to seven times daily, 19% (*N* = 12) three to four times daily and 39% (*N* = 25) one to two times daily. ^i^ *N* = 178. ^j^ *N* = 354. ^k^McNemar test considering patients with paired observations only.

### 4.1 Findings related to previous catheter

A total of 179 patients had previous experience with intermittent catheterization. Of those, 86 patients (48%) had previously used LoFric catheters (Wellspect HealthCare, DENTSPLY IH AB, Sweden), 70 patients (39%) used SpeediCath/EasiCath catheters (Coloplast A/S, Denmark), and 23 patients (13%) used other brands (VaPro, Teleflex, Hydrosil, Actreen, and iQCath). Most patients used a Nelaton catheter tip (*N* = 156; 88%), Charrière size 12 or 14 (*N* = 162; 91%), and 40 cm length (*N* = 159; 89%). A majority of users found their previous catheter easy to use and 79% (*N* = 141) were satisfied with it. It was noted that 32% (*N* = 57) touched the coated part of their previous catheter during catheterization and could potentially benefit from an insertion grip (Table 2).

### 4.2 Findings related to the hydrophilic‐coated catheter

A total of 365 patients returned the second questionnaire, a response rate of 88%. Of those individuals, 72% (*N* = 261) were still using the hydrophilic‐coated catheter. The remaining 28% (*N* = 104) had discontinued their use either instantaneously after less than 1 week (*N* = 16; 15%), after 1–2 weeks (*N* = 20; 19%), or after 3–7 weeks of use (*N* = 61; 59%). The reasons for discontinuation among the 28% who discontinued were no persistent need to catheterize (*N* = 24; 7%), a therapy switch (*N* = 6; 2%), a catheter switch (*N* = 69; 19%), or other reasons (*N* = 5; 1%). The reported reasons for catheter switch were due to preference (*N* = 46; 13%), a supply shortage at the pharmacy (*N* = 8; 2%), complications (*N* = 12; 3%), or advice from a healthcare professional (*N* = 3, 1%). Adequate training before starting with LoFric Origo was reported by 94% (*N* = 335/358) and 6% (*N* = 23/358) reported that this was lacking. See details in Table 2.

Patients evaluating the hydrophilic catheter showed a general satisfaction rate of 81% (*N* = 289/359). Satisfaction rate was also statistically associated with adherence. That is, among those still on the hydrophilic catheter, 99% (*N* = 257/260) reported satisfaction. This can be compared with those who abandon the hydrophilic catheter, who reported a satisfaction of 64% (*N* = 63/90), (*p* < 0.001 [Wilcoxon rank sum test]). New users, with previous experience (up to 3 months), were generally more satisfied (*N* = 202/223; 91%) with the hydrophilic‐coated catheter as compared with users with more than 3 months’ experience (*N* = 87/136; 64%; *p* < 0.001 [Wilcoxon rank sum test]). Most patients (*N* = 305/358; 85%) perceived the hydrophilic‐coated catheter as hygienic due to the insertion grip. The foldable feature was deemed as important by 67% (*N* = 236/351) and 89% (*N* = 309/349) thought that the catheter had an appealing design. The result was that 85% (*N* = 298/351) would recommend the hydrophilic‐coated catheter to a friend and 77% (*N* = 271/354) would like to continue using it. When patients were asked to list the attributes best describing the hydrophilic‐coated catheter from a list of 19 features the following three were most common:

- Insertion aid—hygienic
- Ready to use
- Foldable—easy to carry

All features are listed in the questionnaires provided in supplementary materials.

When comparing the results reported for the previous catheter to the hydrophilic‐coated catheter, similar values were reported for satisfaction (79% vs. 81%; *p*‐value = 0.5657 [McNemar test]), ease of use at insertion (78% vs. 83%; *p*‐value = 0.0886 [McNemar test]), and withdrawal (92% vs. 92%; *p*‐value = 0.3261 [McNemar test]). See details in Table 2.

## 5 DISCUSSION

The results from this study show that real‐life use of the evaluated hydrophilic‐coated catheter seems to work well since the general perception by patients was positive and high satisfaction was reported. Patient satisfaction scores seem to be related to adherence as there was a statistically significant higher satisfaction (99%) among those still on the hydrophilic catheter compared with those who abandon the hydrophilic catheter (64% satisfaction). The general adherence in this study was 72% after 8 weeks. General adherence to intermittent catheterization has been reported to be about 50% after 1 year (Cobussen‐Boekhorst, Beekman, et al., 2016), but this is very much dependent on the studied population. It is difficult to draw specific causality conclusions on satisfaction and adherence associated with specific features of the hydrophilic‐coated catheter. Patients, however, acknowledged the use of the insertion grip on the hydrophilic‐coated catheter and it was seen as a hygienic measure, enabling nontouch catheterization. Furthermore, a total of 81% found the hydrophilic‐coated catheter easy to use.

When comparing the results reported from patients with previous catheter experience to the results reported from everyone who evaluated the hydrophilic‐coated catheter, similar values were reported related to satisfaction, ease of use at insertion, and withdrawal. When considering these results it should be noted that the group evaluating the hydrophilic‐coated catheter also comprised new users, in contrast to the group of previous catheter users. New users may still be in the process of accepting their illness and learning to practice intermittent catheterization (Nevedal, Kratz, & Tate, 2016; Shaw & Logan, 2013). These factors could potentially affect the results in favour of users with previous catheter experience. However, new users were found to be generally more satisfied with the hydrophilic‐coated catheter, as compared with experienced users and this suggests that habit and experience as such are more important factors determining general perception. The results imply that experienced users are less willing to change catheters and, if there are no complications, prefer to stick to the catheter they first started with. It may also reflect that not a single catheter choice suits everybody and individual needs and preferences differ between catheter users. That is, the experienced users in this study may have other catheter preferences than the features provided by the hydrophilic‐coated catheter. Another possible explanation for switching catheters is selective insurance coverage since this sometimes restricts a patient's preferred choice (e.g., the reimbursement level may only cover low‐priced catheters).

The results related to the hydrophilic‐coated catheter confirm previous reports that describe general patient‐preferred catheter features and add more specific details on each general attribute. Portability of the catheter (i.e., easy to carry), hygiene, and readiness for use were all reported as important features of the evaluated hydrophilic‐coated catheter and this is in line with previous literature (Chartier‐Kastler et al., 2013; Neovius & Lundqvist, 2014; Pinder et al., 2015), but it is also more specific. For example, Pinder et al. (Pinder et al., 2015) and Neovius and Lundqvist (Neovius & Lundqvist, 2014) highlight infection prevention as the most important general feature for catheters. In the current study, the hygienic insertion grip of the hydrophilic‐coated catheter was experienced as important by 85% of the users. Furthermore, Pinder et al. (Pinder et al., 2015) describe ease of insertion and convenience as important catheter features. Both of these are confirmed by patients in the current study and convenience in this study is translated into more specific features such as catheter's easy portability and the fact that it is ready to use. Chartier‐Kastler et al., (2013) also conclude that a small catheter design increases quality of life among patients practicing intermittent catheterization. The results from the current study seem to verify this conclusion as 89% found the slim catheter design of the hydrophilic‐coated catheter appealing and 67% found the foldable feature of the catheter to be important.

### 5.1 Limitations

Any observational study has its inherent limitations related to the design. For example, causality relationships are compromised by the fact that it is not possible to control factors having an impact on the outcome. Observational research is, however, a useful way to observe real‐life situations and to understand current clinical practice. It should be noted that in the current study no financial incentives were influencing the patients’ preferences or opinions (i.e., no free catheter samples were given in addition to those which were part of normal routines) and that is why the results are considered to reflect “real world data” and normal routines associated with intermittent catheterization.

Another limitation of the study was the use of a nonvalidated patient‐reported questionnaire for collection of the study variables. The selected questions were, however, based on previous findings from studies (e.g., (Johansson et al., 2013)), experiences from product development, and clinical expertise. It is acknowledged that since the time point of the study, validated forms have been published that might have been suitable for this study. Full questionnaires can be reviewed in supplementary material.

As with any survey, the current study is compromised by some patients who are lost during follow‐up and data that are missing. The reasons for drop‐out are not known but the overall response rate (88%) is higher than other surveys of similar populations where 23% (Woodbury et al., 2008) and 56% (Hakansson et al., 2015) are reported. Even though the overall response rate seems high, the completeness of each individual questionnaire was not ideal as patients felt that they were not able to answer all specific questions. To limit biases, missing data were not computed or replaced and percentages presented were based on available answers only. Finally, it is acknowledged that the respondents were self‐selecting and it is impossible to say if they constitute a representative sample of the whole population applicable for intermittent catheterization. However, the generous inclusion criteria and the substantial total number of patients provide a good basis for the generalizability of the results.

## 6 CONCLUSIONS

Patients reported a high level of satisfaction and adherence to the evaluated hydrophilic‐coated catheter. Patients acknowledged the usefulness of the hygienic insertion grip of the hydrophilic‐coated catheter, enabling nontouch catheterization. The results confirm previous reports that describe patient‐preferred catheter features to be convenience, ease of use, and infection prevention. It adds knowledge of preferred and appreciated specific catheter attributes such as portability and that a catheter can be folded, ready to use, and include hygienic measures such as an insertion grip.

### 6.1 Implications for clinical practice

To ensure good adherence of intermittent catheterization, it is important to ensure that patients are given a choice of a convenient and easy to use catheter that minimizes any risk of infection. Good adherence is believed to reduce the risk of complications and to optimize the outcome of the therapy. The results from the current study propose that the hydrophilic‐coated catheter, LoFric Origo, fulfils patient‐preferred catheter requirements and for this reason it seems to be a useful addition to nurses’ arsenal of catheters offered to men who are introduced to intermittent catheterization. In addition, it may be a useful alternative for experienced users of intermittent catheterization who are not satisfied with their current treatment.

## CONFLICTS OF INTEREST

The authors have no specific conflicts of interest to declare. The study was sponsored by Wellspect HealthCare, DENTSPLY IH AB, Sweden, but there was no specific compensation related to its publication.

## AUTHOR CONTRIBUTIONS

IWK: Protocol/project development, data collection and analysis, and manuscript writing. GS, NAH, LRL, HC, DSM, SR, and HJPFA: Data collection, manuscript revision. The LoFric Origo study group: Data collection.

All authors have agreed on the final version and meet at least one of the following criteria [recommended by the ICMJE (https://www.icmje.org/recommendations/)]:

- substantial contributions to conception and design, acquisition of data, or analysis and interpretation of data;
- drafting the article or revising it critically for important intellectual content.
