# Supplementary material for: User perception of a new hydrophilic‐coated male urinary catheter for intermittent use
Source: Nurs Open. 2018 Sep 4;6(1):116–25. doi: 10.1002/nop2.193 (PMC6279713; doi:10.1002/nop2.193)
Supplement: Supplementary file 1 [file NOP2-6-116-s001.pdf]

Questionnaire 1

Diagnosis

1. Please specify the main reason why you need to catheterize. If you are unsure, ask your study physician or study nurse

- ○ Brain and/or spinal cord disease (e.g. Multiple Sclerosis, myelitis, Tumour, cyst)
- ○ Neural tube defects (e.g. Spina Bifida)
- ○ Spinal cord injury, paraplegia
- ○ Spinal cord injury, tetraplegia
- ○ Bladder dysfunction (e.g. underactive detrusor, overactive bladder)
- ○ Bladder outlet obstruction (e.g. prostate hyperplasia)
- ○ Post-surgical condition (e.g. neobladder, orthotopic bladder substitutes)
- ○ Other (specify): ______

______

Start of catheterisation

2. Please specify how long you have practiced intermittent catheterisation

- ○ New user
- ○ < 3 months
- ○ 3 months to 1 year
- ○ 1-3 years
- ○ > 3 years

Hand function

3. Please specify the level of your hand function

- ○ Normal hand function
- ○ Slightly reduced
- ○ Considerably reduced

Urethral sensitivity

4. Please specify what level of sensation you have in your urethra

- ○ Normal urethral sensitivity
- ○ Reduced sensitivity
- ○ No sensitivity at all

If you are a new catheter user, please ignore the remaining questions in the survey

Type of catheter used

5. Please specify which catheter you previously used

- ○ LoFric
- ○ LoFric Primo
- ○ LoFric Hydro-Kit
- ○ SpeediCath
- ○ SpeediCath Compact Male
- ○ EasiCath
- ○ SpeediCath Complete
- ○ Other (specify):

Type of catheter tip

6. Please specify the type of catheter tip

- ○ Nelaton
- ○ Tiemann
- ○ Other (specify):

Years/months using previous catheter

7. Please specify how long you have practiced intermittent catheterisation with your previously used catheter

- ○ < 3 months
- ○ 3 months to 1 year
- ○ 1-3 years
- ○ > 3 years

Number of catheterisations per day

8. Please specify the average number of catheterisations per day

○	○	○	○	○	○	○	○
1	2	3	4	5	6	7	>7

Body position

9. Please specify your typical body position during catheterisation

- ○ Sitting
- ○ Standing
- ○ Lying down

10. Do you catheterize yourself (majority of the time)?

- ○ Yes
- ○ No

Bladder management methods

11. In addition to catheterisation, specify other bladder management methods used to empty bladder. Please tick all that apply

- ○ Not applicable
- ○ Normal voiding with or without straining
- ○ External compression
- ○ Other methods (specify):

Incontinence

12 a) Do you experience involuntary urine loss between catheterisation/ voidings?

- ◯ Yes
- ◯ No

b) If Yes, please specify incontinence aid(s) used

- ◯ Absorbent products (for example shields, under garments, sanitary towels, sheets, diapers etc)
- ◯ External urine collections device (for example condom catheter, urine collection bags, drip collectors etc)
- ◯ Other methods (specify): ____________

Catheter size and length

13. Please specify the Charriere size (CH) and the length (cm) of the previously used catheter

Size (CH): ____________ Length (cm): ____________

Practical to use – at home

14. Is your previously used catheter practical to use at home?

- ◯ Very practical
- ◯ Practical
- ◯ Neutral
- ◯ Not so practical
- ◯ Not practical at all

Practical to use – out of the house

15. Is your previously used catheter practical to use out of the house?

- ◯ Very practical
- ◯ Practical
- ◯ Neutral
- ◯ Not so practical
- ◯ Not practical at all

Handling – before insertion

16. How do you find the handling of your previously used catheter *prior to insertion?*

- ◯ Very easy
- ◯ Easy
- ◯ Neutral
- ◯ Difficult
- ◯ Very difficult

Handling – at insertion

17. How do you find the handling of your previously used catheter *during insertion*?

- ◯ Very easy
- ◯ Easy
- ◯ Neutral
- ◯ Difficult
- ◯ Very difficult

Handling – at withdrawal

18. How do you find the handling of your previously used catheter *at withdrawal*?

- ◯ Very easy
- ◯ Easy
- ◯ Neutral
- ◯ Difficult
- ◯ Very difficult

Handling – after withdrawal

19. *After withdrawal*, do you find the catheter easy to re-insert into the packaging?

- ○ I do not re-insert
- ○ Very easy
- ○ Easy
- ○ Neutral
- ○ Difficult
- ○ Very difficult

20. *After withdrawal*, do you find the catheter easy to dispose of?

- ○ Very easy
- ○ Easy
- ○ Neutral
- ○ Difficult
- ○ Very difficult

Coating

21. During insertion, do you usually touch the coated part of the catheter?

- ○ Yes
- ○ No, I use the insertion aid enclosed with my product
- ○ No, I use a separate insertion device
- ○ No, I hold the connector

Urine bags

22. Did you connect your previously used catheter to urine bags?

- ○ Yes
- ○ Yes, but only sometimes
- ○ No

Extension to your catheter

23. Do you use some type of extension device (e.g. packaging, extra tube etc.)

- ○ Yes
- ○ Yes, but only sometimes
- ○ No

Satisfaction

24. Are you satisfied with your previously used catheter?

- ○ Completely satisfied
- ○ Satisfied
- ○ Neutral
- ○ Not satisfied
- ○ Not satisfied at all

Questionnaire 2

Compliance of LoFric Origo

1.
a) Do you still perform catheterisation with LoFric Origo?

- ○ Yes
- ○ No

b) If No, please specify the reason why:

c) If No, please specify how many weeks you have performed catheterisation with LoFric Origo

○	○	○	○	○	○	○	○
<1 w	1 w	2 w	3 w	4 w	5 w	6 w	7 w

d) Please specify the average number of catheterisations per day during the time you used LoFric Origo

○	○	○	○	○	○	○	○
1	2	3	4	5	6	7	> 7

Perception

2. Please specify how satisfied you are with LoFric Origo

- ○ Completely satisfied
- ○ Satisfied
- ○ Neutral
- ○ Not satisfied
- ○ Not satisfied at all

3.
a) Did you experience any unexpected discomfort when using LoFric Origo?

- ○ Yes
- ○ No

b) If Yes, please specify the type of discomfort. Several answers possible

- ○ Pain
- ○ Burning sensation
- ○ Bleeding
- ○ Other, please specify:

General questions

4. How did you hear about LoFric Origo? Please tick all that apply.

- ○ From my healthcare professional/nurse
- ○ From my caregiver
- ○ From my friends/ family
- ○ From other catheter users
- ○ On LoFric website
- ○ On other website
- ○ Blogs
- ○ Magazine
- ○ Conference/exhibition
- ○ Patient organisation
- ○ Other, please specify:

Body position

5. Please specify your typical body position during catheterisation

- ◯ Sitting
- ◯ Standing
- ◯ Lying down

6. Do you catheterize yourself (majority of the time)?

- ◯ Yes
- ◯ No

Bladder management methods

7. In addition to catheterisation, specify other bladder management methods you use to empty bladder. Please tick all that apply.

- ◯ Not applicable
- ◯ Normal voiding with or without straining
- ◯ External compression
- ◯ Other methods (specify):

Incontinence

8.
a) Do you experience involuntary urine loss between catheterisation/ voidings?

- ◯ Yes
- ◯ No

b) If Yes, please specify incontinence aid(s) used

- ◯ Absorbent products (for example shields, under-garments, sanitary towels, sheets, diapers etc)
- ◯ External urine collections device (for example condom catheter, urine collection bags, sheaths etc)
- ◯ Other methods (specify):

Why LoFric Origo?

9. Why did you choose LoFric Origo? Please tick all that apply.

- ◯ I wanted to test a new catheter
- ◯ Appealing design
- ◯ Discreet
- ◯ Recommendation from healthcare professional
- ◯ Not satisfied with my previous catheter
- ◯ Recommendation from a friend
- ◯ Other, please specify:

Instructions and training

10. Did you receive adequate instructions and training on how to perform catheterisation with LoFric Origo?

- ◯ Yes
- ◯ No

Practical to use – at home

11. Is the LoFric Origo catheter practical to use at home?

- ◯ Very practical
- ◯ Practical
- ◯ Neutral
- ◯ Not so practical
- ◯ Not practical at all

Practical to use – out of the house

12. Is the LoFric Origo catheter practical to use out of the house?

- ◯ Very practical
- ◯ Practical
- ◯ Neutral
- ◯ Not so practical
- ◯ Not practical at all

Handling – before insertion

13. How do you find the handling of LoFric Origo *prior to insertion?*

- ○ Very easy
- ○ Easy
- ○ Neutral
- ○ Difficult
- ○ Very difficult

Handling – at insertion

14. How do you find the handling of LoFric Origo *during insertion?*

- ○ Very easy
- ○ Easy
- ○ Neutral
- ○ Difficult
- ○ Very difficult

Handling – at withdrawal

15. How do you find the handling of LoFric Origo *at withdrawal?*

- ○ Very easy
- ○ Easy
- ○ Neutral
- ○ Difficult
- ○ Very difficult

Handling – after withdrawal

16

a) *After withdrawal*, do you find LoFric Origo easy to re-insert into the packaging?

- ○ I do not re-insert
- ○ Very easy
- ○ Easy
- ○ Neutral
- ○ Difficult
- ○ Very difficult

b) After withdrawal, do you find the catheter easy to dispose of

- ○ Very easy
- ○ Easy
- ○ Neutral
- ○ Difficult
- ○ Very difficult

c) If you find the handling of LoFric Origo difficult, please explain why: ____________________

____

____

Activation

17. How do you find the activation of LoFric Origo (bursting the water sachet)?

- ○ Very easy
- ○ Easy
- ○ Neutral
- ○ Difficult
- ○ Very difficult

Opening

18. How do you find the opening of LoFric Origo?

- Very easy
- Easy
- Neutral
- Difficult
- Very difficult

Hanging function

19. Do you like the hanging function of LoFric Origo (sticker on the back of product)?

- I don't use the hanging function
- Yes
- No, please explain why:

Length

20. Do you find LoFric Origo long enough to ensure complete bladder emptying?

- Yes
- No

Insertion Grip – easy to use

21. Do you find the Insertion Grip easy to use?

- Do not use
- Yes
- No, please explain why:

Insertion Grip – Hygienic

22. Do you find LoFric Origo hygienic because of the Insertion Grip?

- Do not use
- Yes
- No

Insertion Grip – Control of the catheter

23. Do you find that the Insertion Grip gives you sufficient control of the catheter during catheterisation?

- Do not use
- Yes
- No

Folding ability

24.

a) Do you sometimes fold LoFric Origo?

- Yes
- No

b) Is the option to be able to fold LoFric Origo important to you?

- Was not aware of the function
- Yes
- No, please explain why:

Design

25. Does the design of LoFric Origo appeal to you?

- Yes
- No

Recommendation to a friend

26. Would you recommend LoFric Origo to a friend?

- Yes
- No

Continue using LoFric Origo

27. Would you like to continue using LoFric Origo?

- Yes
- No

Your opinion of LoFric Origo?

28. Choose *3 attributes* that you believe *best describes* LoFric Origo. If you are in doubt, choose the attributes that most closely reflect your opinion.

- ○ Foldable – easy to carry
- ○ Foldable – discreet
- ○ Insertion aid – hygienic
- ○ Insertion aid – better control
- ○ Easy to hold catheter
- ○ Easy activation
- ○ Activation before use – always fresh and hygienic
- ○ Instant activation – you are in control
- ○ Discreet design
- ○ Ready to use
- ○ Well-known brand (LoFric)
- ○ PVC free
- ○ Surface (coating) – low friction during insertion/withdrawal
- ○ Surface (coating)
- ○ Discreet disposal
- ○ Hygienic disposal
- ○ Easy to open
- ○ Hanging function
- ○ Long enough (complete bladder emptying)

29. Choose *3 attributes* that you believe *least describes* LoFric Origo. If you are in doubt, choose the attributes that most closely reflect your opinion.

- ○ Foldable – easy to carry
- ○ Foldable – discreet
- ○ Insertion aid – hygienic
- ○ Insertion aid – better control
- ○ Easy to hold catheter
- ○ Easy activation
- ○ Activation before use – always fresh and hygienic
- ○ Instant activation – you are in control
- ○ Discreet design
- ○ Ready to use
- ○ Well-known brand (LoFric)
- ○ PVC free
- ○ Surface (coating) – low friction during insertion/withdrawal
- ○ Surface (coating)
- ○ Discreet disposal
- ○ Hygienic disposal
- ○ Easy to open
- ○ Hanging function
- ○ Long enough (complete bladder emptying)
